# Survival of the feces: Does a nematode lungworm adaptively manipulate the behavior of its cane toad host?

**DOI:** 10.1002/ece3.3870

**Published:** 2018-04-15

**Authors:** Patrick B. Finnerty, Richard Shine, Gregory P. Brown

**Affiliations:** ^1^ School of Life and Environmental Sciences University of Sydney Sydney NSW Australia

**Keywords:** *Bufo marinus*, deworming, extended phenotype, host–parasite manipulation

## Abstract

Parasites can enhance their fitness by modifying the behavior of their hosts in ways that increase rates of production and transmission of parasite larvae. We used an antihelminthic drug to experimentally alter infections of lungworms (*Rhabdias pseudosphaerocephala*) in cane toads (*Rhinella marina*). We then compared subsequent behaviors of dewormed toads versus toads that retained infections. Both in the laboratory and in the field, the presence of parasites induced hosts to select higher body temperatures (thereby increasing rates of lungworm egg production), to defecate in moister sites, and to produce feces with higher moisture content (thereby enhancing survival of larvae shed in feces). Because those behavioral modifications enhance rather than decrease parasite fitness, they are likely to have arisen as adaptive manipulations of host behavior rather than as host adaptations to combat infection or as nonadaptive consequences of infection on host physiology. However, the mechanisms by which lungworms alter cane toad thermal preference and defecation are not known. Although many examples of host manipulation by parasites involve intermediate hosts facilitating their own demise, our findings indicate that manipulation of definitive hosts can be as subtle as when and where to defecate.

## 1 INTRODUCTION

The negative impacts of parasites on their hosts can arise through diverse pathways. Some symptoms of infection are due to physiological or behavioral responses by the host (e.g., fever and upregulation of immune system) that function to kill or damage the pathogen (Kelley, Aubert, & Dantzer, 2012). Alternatively, changes in host physiology or behavior that enhance rather than decrease pathogen proliferation and transmission may indicate a pathogen with the ability to subvert host behavior for its own benefit. Thus, the modification of host behavior by parasites can be shaped by selection either on the host or on the parasite. We can distinguish between these alternatives by looking at whether the parasite is benefited or harmed by the changes in its host's behavior. Parasites that can manipulate the behavior of their hosts—an example of Dawkins’ (1999) “extended phenotype” concept—might thereby leave more offspring than conspecific parasites whose effects on the host were less specific (Hughes, Brodeur, & Thomas, 2012). Given the high species diversity of parasites, and their typically short generation times and high fecundity, host manipulation “tactics” by parasites may evolve rapidly (Poulin, 1995; Thomas et al., 2002).

Numerous examples of metazoan parasites manipulating their hosts have emerged over the past decade (Thomas, Poulin, & Brodeur, 2010). One review of this topic (Dobson, 1988) listed 27 host‐manipulative parasites (trematodes, cestodes, nematodes, and acanthocephalans), all with indirect lifecycles (i.e., involving both an intermediate host and a final host). Just over a decade later, an entire book was filled with examples of parasite taxa manipulating the behavior of their hosts and involving a far broader range of hosts (from small invertebrates to large vertebrates) and in both aquatic and terrestrial ecosystems (Moore, 2002). Many well‐known examples of host–parasite manipulation involve metazoan parasites with indirect lifecycles that manipulate the behavior of an intermediate host in ways that render that animal more likely to be consumed by the definitive host (Adamo, 2012; Lafferty & Kuris, 2012; Poulin, 2010; Schmid‐Hempel, 2011; Thomas et al., 2010). Host manipulation by metazoan parasites with direct lifecycles is less well known (Lafferty & Kuris, 2012).

Many direct‐lifecycle parasites are helminths (Anderson, 1988, 2000; Shoop, 1988), with infective larvae often transferred to new hosts via contact with feces (Anderson, 1988; Craig & Ito, 2007; Mackiewicz, 1988; Shoop, 1988). In contrast to indirect‐lifecycle parasites, direct‐lifecycle parasites would benefit by enhancing rather than decreasing the survival of the definitive host in which they live (Phillips, 2012). Multidimensional manipulation of host behavior in such a system is likely to be subtle; for example, parasite fitness might be enhanced by causing an infected host to become gregarious (thereby increasing rates of transmission) or to select habitats that enhance rates of survival or transmission of parasite larvae (Park & Sparkes, 2017; Perrot‐Minnot, Maddaleno, Balourdet, & Cézilly, 2012; Thomas et al., 2010).

It may sometimes be difficult to distinguish “adaptive” manipulation of host behavior from the simpler explanation of a direct impact of the parasite on host physiology. Ectotherms often respond to infection by selecting higher body temperatures, and this “behavioral fever” can increase host survival and decrease pathogen survival (Rakus, Ronsmans, & Vanderplasschen, 2017). Other cases of generalized “sickness behavior” (e.g., lassitude and lack of social interaction) may be most parsimoniously interpreted as a direct effect of the parasite rather than as a suite of “manipulated” traits—even if that “sickness behavior” confers fitness benefits for the parasite (Barber & Dingemanse, 2010; Klein, 2003; Kortet, Hedrick, & Vainikka, 2010; Perrot‐Minnot et al., 2012; Thomas, Adamo, & Moore, 2005). Nonetheless, some of the host behaviors elicited by parasites are highly specific and clearly enhance transfer to the final host. For example, the fluke *Dicrocoelium dendriticum* induces its intermediate host (a carpenter ant, *Camponotus pennsylvanicus*) to raise its abdomen from the tip of a blade of grass, thereby increasing the chances of a herbivore (the final, definitive host) ingesting the ant (Carney, 1969). Infected hosts are often “deeply modified” in multiple ways that increase the probability of parasite transmission (Brodeur & Boivin, 2004; Cézilly & Perrot‐Minnot, 2005; Cézilly, Thomas, Médoc, & Perrot‐Minnot, 2010; Ponton et al., 2005).

In this paper, we investigate the hypothesis that a direct‐lifecycle parasite can exert multidimensional host manipulation. As a study system, we use the invasive cane toad (*Rhinella marina*) and a direct‐lifecycle nematode, the lungworm *Rhabdias pseudosphaerocephala* (Kelehear, Webb, & Shine, 2009; Pizzatto, Kelehear, & Shine, 2013). What changes in behavior of an infected toad might be targets of selection for host manipulation? Because completion of the *Rhabdias* lifecycle requires moist soil (Koprivnikar et al., 2012; Langford & Janovy, 2016), a parasite that induces its host to deposit feces on a moist substrate might not only ensure that the larvae survive to the infective stage, but also increase its chances of encountering a rehydrating host to infect. Similarly, if the parasite's reproductive rate is temperature‐sensitive, inducing the host to select and maintain a warmer or cooler body temperature might increase *Rhabdias* fitness. To test these predictions, we experimentally modified infection status of toads both in the laboratory and in the wild, and monitored their behavior. To evaluate the impact of such changes on parasite viability, we conducted trials to quantify the impact of host temperature and substrate conditions on the rates of survival of larval lungworms.

## 2 MATERIALS AND METHODS

### 2.1 Study species

Cane toads (*Rhinella marina,* formerly *Bufo marinus*) are large (up to 500 g) toxic bufonid anurans native to South and Central America. Introduced into Australia in 1935, cane toads now inhabit more than one million square kilometers (Phillips, Brown, Webb, & Shine, 2006; Urban, Phillips, Skelly, & Shine, 2008) and have caused population declines of endemic Australian predators (Shine, 2010). Brought to Australia with the originally imported cane toads, *Rhabdias pseudosphaerocephala* are lung nematodes that now occur throughout the toad's Australian range (Dubey & Shine, 2008) except for the invasion front (Phillips et al., 2010). Adult hermaphroditic worms inside the toad's lungs release eggs that move into the host's digestive system and hatch into first‐stage male and female free‐living forms. After the toad defecates, these free‐living larvae mate to produce infective third‐stage larvae (L3) that develop inside their mother for up to 4 days before breaking free and entering the soil (Baker, 1979). When an L3 locates an anuran host, it pierces through the epidermis and migrates through tissue to reach the lungs of the toad where it feeds on blood from capillary beds (Pizzatto, Shilton, & Shine, 2010) and can mature in as few as 5 days (Kelehear, Brown, & Shine, 2012). Although infection dynamics vary seasonally and climatically (Barton, 1998; Pizzatto et al., 2013), the parasite is common in some populations of toads in Australia (>80% of toads infected: Barton, 1998), with up to 282 adult worms per host (Pizzatto et al., 2013).

### 2.2 Study site

We conducted our study between August and November 2016 in the vicinity of Leaning Tree Lagoon, a 6‐ha billabong, 80 km southeast of Darwin in Australia's Northern Territory. The area experiences a wet–dry tropical climate with monsoonal rainfall between November and April (Shine & Brown, 2008). Our study took place primarily during the dry season when average monthly maximum air temperature exceeded 35°C (BOM 2016). Cane toads arrived in the area late in 2005, and lungworms were first recorded in 2008 (Phillips et al., 2010).

### 2.3 Studies on captive toads

Over three nights, we captured 49 toads (mean snout–urostyle length [SUL] ± *SE* = 78.3 ± 12.2 mm) from multiple sites on the Adelaide River floodplain (12.6°S, 131.3°W) and took them back to the laboratory where they were weighed (to the nearest 0.1 g), measured (to the nearest 0.1 mm: SUL), and toe‐clipped for individual identification (Hudson, Brown, & Shine, 2017). Each toad was individually housed in a plastic box of 300 × 200 × 200 mm. Over the next 4 months (August–November 2016), all toads were provided with constant access to water and offered at least four large crickets every second day. To quantify each toad's infection status, its feces were viewed under a dissecting microscope for the presence of *Rhabdias* larvae. Larvae were identified as *R. pseudosphaerocephala* based on their unique shape, size, and movement patterns. No other known cane toad parasites in Australia resemble *Rhabdias pseudosphaerocephala* larvae or have been recorded in cane toad feces (Pizzatto, Kelehear, Dubey, Barton, & Shine, 2012; Pizzatto et al., 2010, 2013). Noninfected and infected toads were randomly assigned to receive either two doses (once at capture and 2 weeks later) of (1) ivermectin (Ivomec ©, Merial Ltd., Duluth, USA) diluted in Amphibian Ringer's solution and administered at a dose of 0.02 mg/100 g toad, or (2) an equivalent volume of Amphibian Ringer's solution alone. Using this method, we generated four treatment groups (ID = infected and dewormed [*n *=* *11 toads], IC = infected and Amphibian Ringer's control [*n *=* *13], ND = not infected and dewormed [*n *=* *13], and NC = not infected and Amphibian Ringer's control [*n *=* *12]). Over the next 4 months, the 49 toads (or subsets thereof) were subjected to a series of behavioral and physiological trials to assess the effects of *Rhabdias* on a range of traits.

#### 2.3.1 Do lungworms affect the thermoregulatory behavior of their hosts?

Toads are nocturnally active and sequester in sheltered refugia during daytime. Shelter sites are selected based on thermal and hydric characteristics (Cohen & Alford, 1996; Tingley & Shine, 2011). We constructed thermal gradients to compare daytime temperature selection among toads in our four treatment groups. All 49 captive toads from the four treatment groups (IC, ID, NC, and ND) were utilized in this component of the study. Large plastic arenas (620 × 420 × 370 mm, 70 L) were placed above heating cables in a temperature‐controlled room set at 18°C. Using 15‐watt cables under one end of the arena and 25‐watt cables under the other, we generated a thermal gradient within each arena with substrate temperatures ranging from 17 to 50°C. Arenas were aligned at the same orientation, and the bottom of each was marked at 155‐mm intervals to divide the floor area into four equal sections. We attached a thermal data logger (Thermochron iButton, Dallas Semiconductor, Texas, USA) in the middle of each section using silicone. The data loggers recorded temperature at 5‐min intervals, and we validated their accuracy by also measuring temperatures at the logger locations with an infrared thermometer (simple linear regression showed infrared thermometer readings to be highly correlated with the readings from iButton data loggers: *F*_1,46_ = 10,562.07, *p *<* *.01, *R* ^2^ **= .99). Moist paper towel covered the base of the arena to maintain an equal moisture level throughout and was replaced after each trial. Before each toad was placed into an arena, we recorded its initial body temperature using an infrared thermometer (Digitech, QM7221, accuracy ±1%) held 10 mm from and aimed at the middle of the toad's dorsum (Karavlan & Venesky, 2016). Each toad was individually placed into the middle of an arena and filmed for 2 hr to quantify the amount of time spent in each quadrat. When a trial concluded, we recorded what quadrat the toad was in and remeasured the toad's body temperature. Trials were conducted during daytime, in a dimly lit room.

#### 2.3.2 Does host temperature influence rate of production of lungworm larvae?

For this component of the study, we used 10 of the 13 toads in the IC group. Ten individuals with similar larval counts in their feces were randomly allocated to “cold” versus “hot” treatments (*n *=* *5 toads per treatment). Toads were kept individually in 12‐L plastic boxes, lined with dry newspaper flooring and a dish of water in a room maintained at 16.1 ± 0.02°C in the “cold” treatment (average humidity 51%) and at 34.1 ± 0.03°C for the “hot” treatment (average humidity 78%). Hourly room temperatures were recorded using thermal data loggers. After 3 days, treatments were switched: The “cold treatment” toads were placed into the “hot treatment” for a further 3 days and vice versa. Before the trial began, each toad was fed four large adult crickets. Each time a toad defecated, we recorded the date, time, and toad body temperature (using an infrared thermometer). We removed two small (0.25 g) subsamples from each fecal pellet (one from either end, counts of *R. pseudosphaerocephala* larvae were similar on both sides of the same fecal mass and highly correlated: *F*_1,24_ = 78.16, *p *<* *.01, *R* ^2^ = .77), mixed them with 1 ml of water, and observed them under a dissection microscope to count larvae in each sample. We used average fecal larval counts for each toad as the dependant variable in the analyses.

#### 2.3.3 Do lungworms affect hydration behavior of toads under hot or cold conditions?

To test whether ambient temperature affected the amount of time spent in the water by infected versus dewormed hosts, we randomly selected 10 infected (IC) and 10 dewormed (ID) captive adult cane toads and placed them into either “cold” (*n *=* *10, mean temperature 16.14 ± 0.02°C, average humidity 49%) or “hot” (*n *=* *10, mean temperature 33.05 ± 0.01°C, average humidity 77%) rooms. Room air temperature was recorded every 10 min using a thermal data logger. Each toad was housed individually in a 12‐L plastic box, lined with dry newspaper and with ad libitum access to water (in a plastic dish). The surface area of the water dish occupied 17% of the floor area (150 cm^2^ of 880 cm^2^). Over the next 3 days, we measured each toad's body temperature twice a day using an infrared thermometer. An “Infrared ScoutGuard Zero Glow 10 m” (Molendinar, Queensland, Australia) camera 2 m above all boxes took a photograph every 10 min. After 3 days, treatment groups were switched with “cold treatment” toads being transferred to hot conditions and vice versa for another 72 hr. To explore any difference between infected and dewormed toads in the time spent in water, we scored the number of times each toad was photographed sitting in the water dish, versus on the dry newspaper, over 72 hr.

#### 2.3.4 Do lungworms influence rates of water loss by their hosts?

We randomly selected nine of the 13 infected (IC) and nine of the 11 dewormed (ID) captive toads and placed them in individual desiccating environments for 24 hr. Each toad was placed in a 5‐L clear plastic container with a mesh lid and two large mesh inserts on either side to allow airflow. All toads were fed four large crickets, weighed, and then placed in the individual containers at 32.5 ± 0.03°C for 24 hr. Toads were weighed every 2 hr until a 20% mass loss (lethal evaporative mass loss in toads is approximately 30%) was recorded, at which time the toad was immediately placed back into its home enclosure to rehydrate. After 24 hr, all remaining toads (that had not yet reached 20% dehydration) were weighed and percentage mass loss was calculated. We used percentage mass loss per hour as the dependent variable in our analyses.

#### 2.3.5 Do lungworms affect the spatial location of defecation by their hosts in captivity?

To determine whether toads in the four treatment groups (IC, ID, NC, and ND) chose different areas in which to defecate, we monitored locations of the feces deposited by all 49 toads over a two‐week period. Toads were held in 12‐L plastic containers lined with dry newspaper (replaced whenever the paper became moist) containing a dish of water (diameter 100 mm). Each toad was fed four large crickets every second day. Four times a day (0800, 1100, 1400, and 1700 hr), we checked to see whether the toad had defecated in its cage and scored the location of the feces as either on the dry newspaper versus in the water. Any feces were immediately removed, and both water and newspaper replaced.

#### 2.3.6 Do lungworms affect the water content of feces produced by their hosts?

Over the 9 days, we collected a total of 29 freshly deposited fecal samples from the 13 infected (IC) and 11 dewormed (ID) captive toads. Cages were checked hourly from 0900 to 1800 hr, and fresh feces were placed onto a 40 × 40 mm square of paper towel and weighed (± 0.01 g). Fecal samples were kept at 23.5 ± 1.4°C (average humidity 52%) and reweighed daily until they ceased losing mass (1.5 days). Initial water content was calculated from the difference between the initial and final masses.

#### 2.3.7 Do hydric conditions affect rates of survival of lungworm larvae?

Freshly deposited fecal samples (*n *=* *8) were removed from the containers of captive infected toads (IC). Each sample was then divided in two and weighed, and each half was placed on top of 60 g of sand (previously heat‐treated at 250°C for 45 min to sterilize it) in a 25‐ml open plastic vial. Sand in one vial was kept damp (at constant weight), whereas the other remained dry. Vials were kept at 23.5 ± 1.4°C (average humidity 52%) for 3 days and then reweighed, and a subsample of fecal matter from each vial was mixed with 1.5 ml of water and observed under a dissection microscope for the presence of lungworm larvae. We counted numbers of larvae in the feces and also in a 5 g sample of sand taken from directly beneath each fecal sample. Results were standardized to number of lungworm larvae per gram of fecal matter and per gram of sand.

### 2.4 Measurement of infection intensity

Four toads (all in the IC group) died during the period of captivity. We dissected the lungs of three of these individuals to count the number of infecting worms. At the end of the study, the remaining captive toads (*n *=* *45) were euthanized with a 0.3‐ml overdose of pentobarbital, then weighed, and dissected. Each lung was removed from the bronchus and inverted over an index finger. All lungworms were counted so that we could compare the effects of infection intensity (number of lungworms) on traits measured during the prior experiments.

Our deworming protocol had the desired effect of virtually eliminating parasites from the lungs of treated toads. At the end of the experiment, nine of the 11 infected toads that were dewormed (ID) had no parasites in their lungs. One toad had a single immature worm in its lungs, and another had two immature worms. All 12 of the uninfected toads that were dewormed (ND) were parasite‐free at the end of the experiment, and one individual had a single parasite. Of the uninfected control toads (NC), nine were parasite‐free, one individual had one worm, and two toads each had two worms. All of the infected control toads (IC) were infected with 4–42 (median = 20) lungworms. Thus, the IC group exhibited significantly higher infection prevalence (χ^2^ = 35.24, *p* < .0001) and intensity (*F*_3,44_ = 32.08, *p* < .0001) than the other three groups.

### 2.5 Studies on free‐ranging toads

We conducted an intensive mark–recapture study at Leaning Tree Lagoon as part of an investigation into the fitness costs associated with *Rhabdias* infection in cane toads (Finnerty, Shine, R., & Brown, G. P., 2017). Over the 3 months, a total of 455 toads were captured and individually marked by toe‐clipping. To determine the infection status of each toad at its initial capture, we held each animal overnight in a 1‐L plastic container with 1 ml of water. For animals that had defecated by the next morning (not all toads defecated), a 1 g of fecal sample was mixed with 1.5 ml of water and viewed under a dissection microscope to check for the presence of *Rhabdias* larvae.

In this manner, we were able to determine the infection status of 123 toads at the time of their first capture. As in the study of captive toads, based on their initial infection status, approximately half of the infected and uninfected toads were treated with ivermectin and the remainder given a control dose of Amphibian Ringer's solution. This treatment regime generated the same four treatment levels as in the study on captive toads (ID, IC, ND, and NC; see above). Toads were released at their capture location within 24 hr. Each time a toad was recaptured, it was remeasured and redosed with the same treatment it received originally and we attempted to collect another fecal sample from it by holding overnight in a 1‐L container (see above).

For the present study, we used a subsample of 82 of the 123 toads. Over 31 nights, we collected 82 of these toads: 40 from the IC group (infected, dosed with Amphibian Ringer's solution) and 42 from the ID group (infected, dosed with ivermectin). Counting the number of larvae in fecal samples produced by these animals enabled us to verify that ivermectin treatment had reduced or eliminated worms from treated toads in the ID group and to ensure that all toads in the IC group were shedding larvae at similar rates.

#### 2.5.1 Do lungworms affect where their hosts defecate in the wild?

We used a modification of the method described by Langford (2010) for detection of amphibian feces in the field. After the infection status of each free‐ranging toad was determined and Ivomec or Amphibian Ringer's solution administered to form the two treatment groups (“infected” IC or “uninfected” ID), each newly captured toad was fed a 0.10‐ml dose of nondigestible, nontoxic UV fluorescent powder (Radglo RPCF; Radiant Color, Belgium) mixed with water (0.12 mg/ml) through a 1‐ml syringe. Toads that remained infected with *Rhabdias* (IC) were fed green powder, whereas dewormed toads were fed yellow powder (so that we could determine the treatment group of feces found in the field). To minimize any bias associated with visibility of different colors of fluorescent powder in the field, we switched infected toads from green to orange powder after the first 2 weeks and switched noninfected toads from yellow to pink powder. As a precaution, we also conducted trials to verify that all colors were equally detectable under field conditions (see Supporting Information).

That night, each toad was released at its site of capture. The following night, using a handheld UV lamp, we located feces from these toads and collected a soil sample directly next to each fecal mass. A handheld GPS waypoint (Garmin, eTrex 10) was recorded at every fecal collection point, enabling us to measure distance to water. The soil samples were taken back to the laboratory and reweighed to constant mass (thus providing a measure of water content of soil at the site of fecal deposition).

#### 2.5.2 How rapidly do toad feces desiccate in the wild?

We used agar models of cane toads and their feces to measure desiccation rates in the microhabitats in which they were found in the wild (see Supporting Information for construction and calibration of agar models). When toad feces were located in the field (using the fluorescent dye technique), we recorded their location (as above) and then removed them and replaced them with an agar model (methods described above). A soil sample from next to the feces was taken back to the laboratory where it was weighed and reweighed to determine water content (as above). The following night, the agar fecal models were returned to the laboratory (in individual sealed bags) to calculate percentage mass loss over the period of deployment in the field.

#### 2.5.3 Radiotelemetry

Beginning in November 2016, after the mark–recapture study at Leaning Tree Lagoon had been under way for 3 months, we radio‐tracked two separate samples of previously marked toads over two “periods” of 5 days, with 2 days separating each “period. “For this component of the study, we only utilized 23 toads that were known at earlier captures to be naturally infected with *Rhabdias* (IC) or to have been naturally infected and then dewormed (ID). Over the first five‐day period, we tracked six toads that had been dewormed (ID) and another six toads that remained infected (IC). Over the second five‐day period, we tracked five toads that had been dewormed (ID) and another six infected toads (IC). Each toad was fitted with a waist belt (made from bead chain) holding a 2‐g transmitter (total mass <3 g: PD‐2, Holohil Systems Ltd., Ottawa, Canada). All toads were adults with SUL >80 mm (mean ± *SE* = 112.4 ± 7.2 mm) and mass from 76 to 194 g; the transmitter and attachments weighed <10% of total body mass (see Richards et al. 1994).

The next morning, toads were released at their original point of capture. After 24 hr, we used a receiver and antenna (Australis 26K, Titley Electronics, Queensland, Australia) to locate each radio‐tagged toad in its diurnal refugium and the GPS coordinates of its location were recorded. An agar block approximating the volume of a toad (toad desiccation rate model) with embedded thermal data logger was placed as close as possible to the toad without disturbing it and left in place for 24 hr. The following morning, the agar block was retrieved, reweighed, and temperature data downloaded. A new agar block was placed beside the toad at its current location (in some cases, at the same site as before). The process continued for four consecutive nights. On the fifth day, transmitter belts were removed.

Weather conditions changed dramatically between the first and second telemetry periods: 158 mm of rain fell at the study site during the second period. Prior to that rainfall, no rain had been recorded at the site for 45 days (BOM 2016). This change in weather allowed us to compare toad behavior during dry weather versus rainy conditions.

### 2.6 Analyses

We used a combination of parametric and nonparametric tests for data analysis. We used Kruskal–Wallis nonparametric tests to compare measures among groups of toads when data were not normally distributed. We used ANOVAs and simple linear regressions to detect differences and trends in normally distributed data. When significant differences existed among groups, we performed post hoc tests to locate those differences (reported using alphabetical superscript in figures). In cases where multiple observations were collected from individual toads, we either calculated mean values per individual or performed mixed‐model analyses, including individual identity as a random effect. All analyses were performed using JMP Pro 11 (SAS Institute, Cary, NC, USA), and we inspected residuals from parametric tests to ensure the data met the assumptions of normality and homogeneity of variance. The text reports mean values and associated standard errors.

## 3 RESULTS

### 3.1 Studies on captive toads

#### 3.1.1 Do lungworms affect the thermoregulatory behavior of their hosts?

As we had intended, mean temperatures differed among the four quadrats of the thermal gradient arenas (Quadrat A = 49 ± 4.5°C, Quadrat B = 29 ± 3.2°C, Quadrat C = 21 ± 2.1°C, and Quadrat D = 17 ± 1.8°C; ANOVA *F*_3,183_ = 4,709.73, *p *<* *.01; post hoc A > B > C > D). Mean body temperatures of toads did not differ significantly among treatment groups before trials (Kruskal–Wallis χ^2^ = 1.89, *df* = 3, *p *=* *.59) but during the trial infected toads selected higher temperatures (IC, 31 ± 1.1°C) than did noninfected and/or dewormed conspecifics (20.3 ± 1.8°C, Kruskal–Wallis χ^2^ = 22.49, *df* = 3, *p *<* *.01, post hoc IC > ID = NC = ND).

Infected toads (IC) also spent more time in the hottest quadrat (A, mean time = 63.4 ± 12.2 min) than did noninfected and/or dewormed toads (14.5 ± 6.6, Kruskal–Wallis χ^2^ = 15.32, *df* = 3, *p *<* *.01, post hoc IC > ID = NC = ND) and thus less time in the coolest quadrat (D, infected mean = 19.3 ± 8.7, noninfected = 64.3 ± 10.4 *SE*, post hoc IC < ID = NC = ND). There was no significant difference between infected versus noninfected toads in times spent in quadrats B (Kruskal–Wallis χ^2^ = 4.14, *df* = 3, *p *=* *.24) or C (Kruskal–Wallis χ^2^ = 1.43, *df* = 3, *p *=* *.69; post hoc IC = ID = NC = ND). Based on the postmortem determination of infection intensities, anurans with more lungworms had higher body temperatures at the end of trials (*F*_1,14_ = 19.17, *p *<* *.01, *R* ^2^ = .65) and spent more time in the warmest quadrat (*F*_1,14_ = 7.74, *p *=* *.01, *R* ^2^ = .63) and less time in coolest quadrat (*F*_1,14_ = 9.11, *p *<* *.01, *R* ^2^ = .63).

#### 3.1.2 Does toad temperature influence rate of production of larvae?

Feces of infected toads (IC) contained 27% more larvae (162 ± 9.22 larvae per g of feces) when they were held in hot conditions (mean body temperature = 31 ± 1.2°C) than when they were kept cool (16 ± 1.1°C; larval production 115 ± 10.30 per g, ANOVA *F*_1,18_ = 33.96, *p *<* *.01). Infection intensity was positively correlated with larval production under hot conditions (*F*_1,36_ = 7.62, *p *=* *.02, *R* ^2^ = .75) but not under cold conditions (*F*_1,36_ = 0.81, *p *=* *.42).

#### 3.1.3 Do lungworms affect hydration behavior of toads?

**Figure 1:**
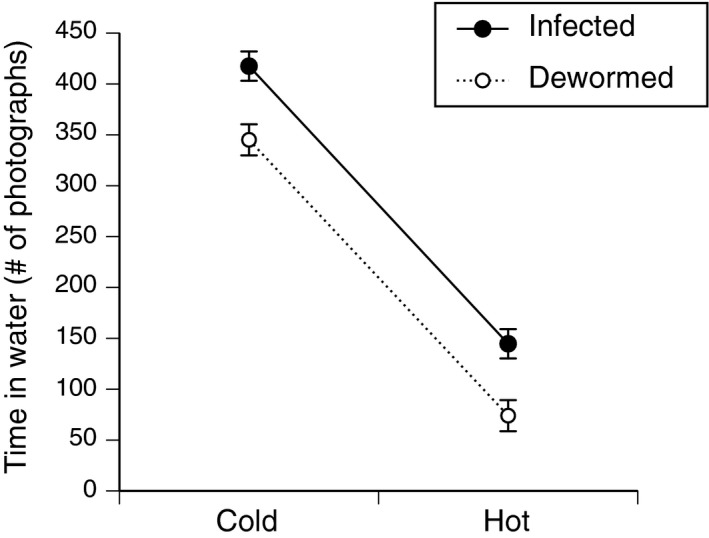
Effects of *Rhabdias pseudosphaerocephala* on the time spent in water by adult cane toads (*Rhinella marina*) maintained under hot (30.07 ± 0.03°C) or cold treatments (16.13 ± 0.02°C). Photographs were taken every 10 min over a 72‐hr period. Graph shows mean values ± 1 *SE*

Overall, infected captive toads (IC) spent more time in the water than did dewormed captive toads (ID) in both cold (16.1 ± 0.02°C) and hot (30.1 ± 0.03°C) treatments (Table 1, Figure 1). Among the infected toads, infection intensity (number of lungworms) was not related to the amount of time spent in water (*F*_1,7_ = 2.75, *p *=* *.14).

**Table 1:** Summary of mixed‐model analyses on the effects of lungworm (*Rhabdias*) infection status (infected IC vs. dewormed ID) on the hydration behavior of 20 captive cane toads and the refugia site selection of 23 radio‐tagged free‐ranging cane toads (*Rhinella marina*). See the text for details. Significant values (*p *<* *.05) are shown in boldface font

Test	Dependent variable	Independent variable	*df*	*F*	*p*
*Hydration behavior of captive toads*					
	Time spent in water	Infection status	1,15	27.41	**<.01**
		Temperature	1,15	291.34	**<.01**
		Infection status × Temperature	1,15	<0.01	.95
*Refuge selection during dry conditions*					
	Distance from water	Infection status	1,11	6.49	**.02**
	Agar model water loss	Infection status	1,11	8.84	**.01**
	Refugia temperature mean	Infection status	1,11	10.28	**<.01**
	Refugia temperature CV	Infection status	1,11	16.07	**<.01**
*Refuge selection during wet conditions*					
	Distance from water	Infection status	1,9	0.04	.85
	Agar model water loss	Infection status	1,9	1.16	.31
	Refugia temperature mean	Infection status	1,9	2.11	.18
	Refugia temperature CV	Infection status	1,9	3.32	.1

“Infection status” refers to two treatment groups of toads: “infected dewormed” ID or “infected” IC. CV = coefficient of variation.

#### 3.1.4 Do lungworms influence rates of water loss by their hosts?

After 24 hr in a desiccation chamber, the mean change in body mass did not differ significantly between infected (IC) and dewormed (ID) captive toads (% mass loss = 18.6 ± 1.2%, *n *=* *9 vs. 18.3 ± 0.8%, *n *=* *9; ANOVA *F*_1,17_ = 0.19, *p *=* *.67). Among the infected toads, the number of lungworms was not significantly associated with desiccation rates (*F*_1,8_ = 1.95, *p *=* *.21).

#### 3.1.5 Do lungworms affect the spatial location of defecation by their hosts in captivity?

Over a nine‐day period, infected toads (IC) defecated more often (mean number of fecal samples/individual = 3.4 ± 0.42) than did uninfected or dewormed toads (ID, NC, ND, 2.1 ± 0.23, Kruskal–Wallis χ^2^ = 7.11, *df* = 1, *p *<* *.01) and were more likely to deposit their feces in their water than on the dry newspaper floor (83% vs. 26%, χ^2^ = 22.7, *df* = 1, *p *<* *.01). Among the infected toads, infection intensity was not significantly associated with the mass of feces produced (*F*_1,14_ = 0.50, *p *=* *.49) nor the location of feces in the cage (χ^2^ = 1.40, *df* = 1, *p *=* *.23).

#### 3.1.6 Do lungworms affect the water content of feces produced by their hosts?

The wet mass of feces produced by infected captive toads (IC) was higher than that of feces from dewormed toads (ID; 0.94 g v. 0.85 g, *F*_1,28_ = 5.23, *p *=* *.03). However, the dry mass of feces did not differ significantly between infected versus dewormed toads (0.41 g v 0.40 g, ANOVA *F*_1,28_ = 1.00, *p *=* *.32). Thus, feces from infected toads had a 15% higher water content (ANOVA *F*_1,28_ = 13.80, *p *<* *.01). Among infected toads, infection intensity did not significantly affect wet mass of the fecal sample (*F*_1,9_ = 1.29, *p *=* *.28), but more heavily infected toads produced feces with a higher water content (*F*_1,9_ = 12.98, *p *<* *.01).

#### 3.1.7 Do hydric conditions affect rates of survival of lungworm larvae?

After 3 days, fewer lungworm larvae survived on dry soil (12 ± 2 larvae/g feces) than on moist soil (183 ± 43 larvae/g feces, paired *t* test, *t *=* *5.8, *df* = 7, *p *<* *.01). Larval numbers were also higher in the sand beneath feces in the moist treatment (8.3 ± 2 larvae/g sand) than in the dry treatment (0 larvae/g sand; paired *t* test, *t *=* *5.80, *df* = 7, *p *<* *.01).

### 3.2 Studies on Free‐ranging toads

#### 3.2.1 Do lungworms affect where their hosts defecate in the wild?

**Figure 2:**
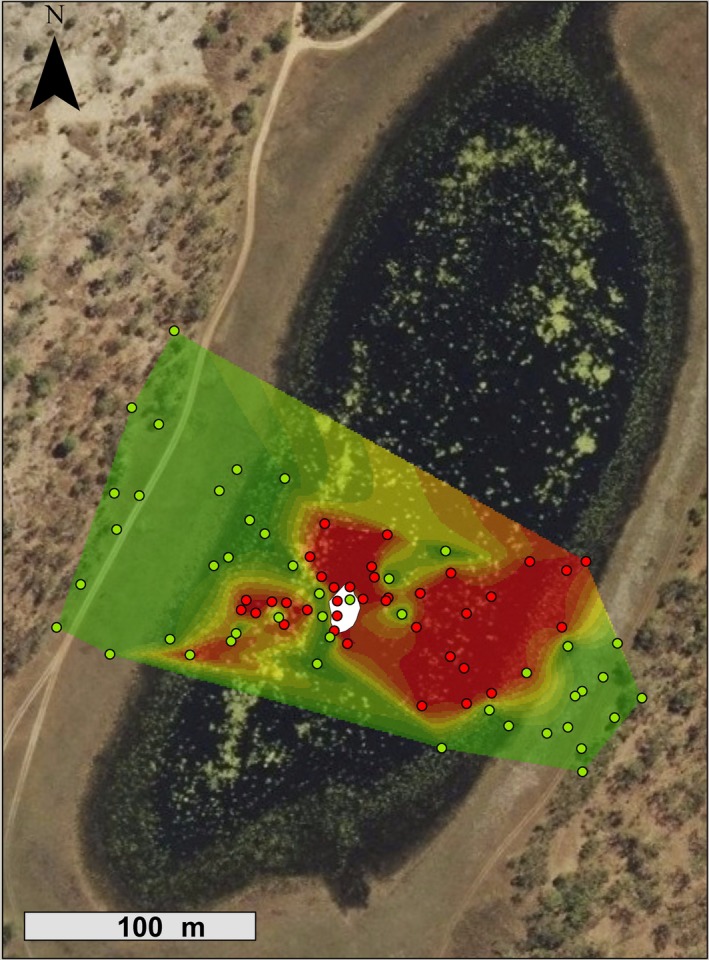
Heat map of the study site showing the effects of *Rhabdias pseudosphaerocephala* infection on the defecation locations of free‐ranging cane toads (*Rhinella marina*). Red points indicate locations of feces from infected toads, and green points indicate feces from dewormed toads. Red areas have a higher probability of containing feces from infected toads. Infected toads defecated closer to the waterbody and on moister soil than did dewormed toads. The central white area indicates water level at the time of the study

Free‐ranging toads that retained natural *Rhabdias* infections defecated closer to the waterbody (IC, 14.2 ± 3.4 m) than did individuals that had been dewormed (ID, 36.4 ± 8.7 m, Kruskal–Wallis χ^2^ = 8.86, *df* = 1, *p *<* *.01; Figure 2). Infected toads also defecated on moister soil than did toads that had been dewormed (16.7 ± 2.1% soil water loss v. 7.6 ± 1.9% soil water loss, Kruskal–Wallis χ^2^ = 18.25, *df* = 1, *p *<* *.01).

#### 3.2.2 How rapidly do toad feces desiccate in the wild?

**Figure 3:**
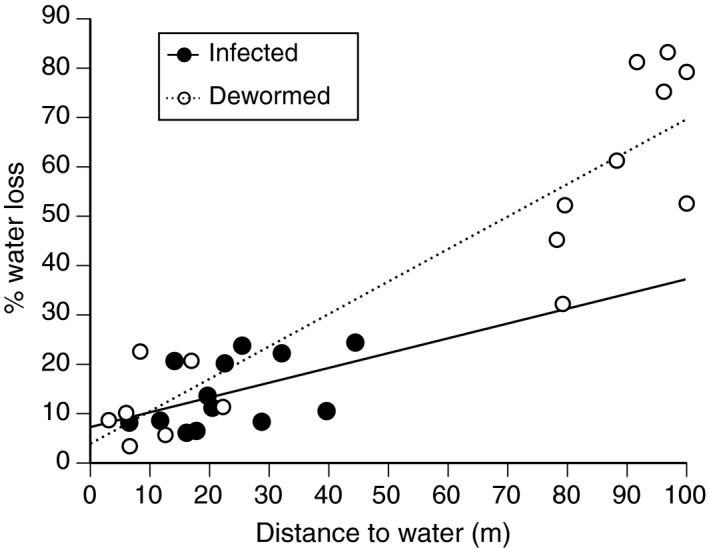
Desiccation rates of agar models in defecation sites used by cane toads infected with *Rhabdias pseudosphaerocephala* (black circles) or dewormed (white circles) in relation to distance from water

Rates of water loss of agar models placed beside feces in the field were higher in areas further from water (*R* ^2^ = .65, *F*_1,28_ = 50.20, *p *<* *.01; Figure 3). Agar models lost water less rapidly when placed at the site where infected toads had defecated than where dewormed toads had defecated (IC vs. ID, Kruskal–Wallis χ^2^ = 7.02, *df* = 1, *p *<* *.01; Figure 3).

#### 3.2.3 Do lungworms affect the types of refuges used by their hosts in the wild?

**Figure 4:**
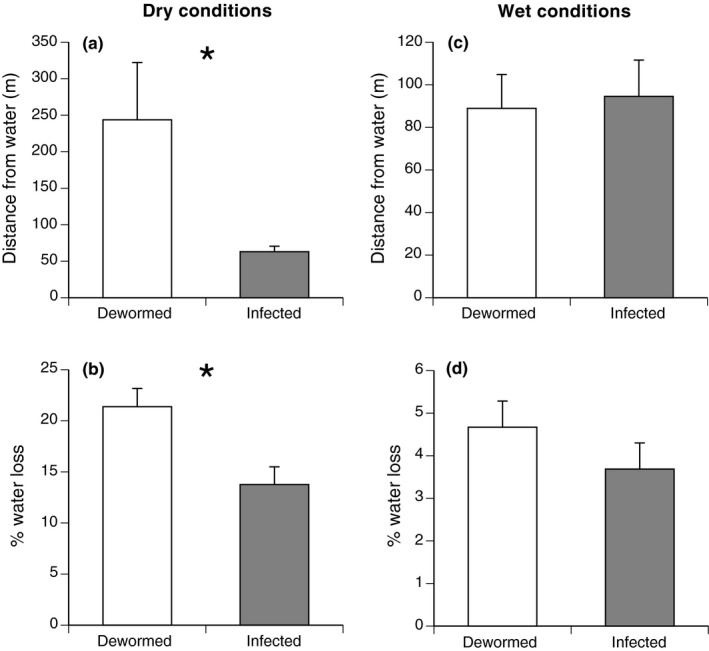
Comparison of characteristics of refuge sites used by 23 radio‐tagged cane toads initially infected with *Rhabdias pseudosphaerocephala,* 12 of which had been subsequently dewormed. Under dry conditions, the six infected toads used refugia that were closer to the water (a) and moister (b) than those used by six dewormed toads. Under wet conditions following a period of heavy rainfall, the five infected toads and six dewormed toads were found in refugia at similar distances from water (c) and with similar desiccation rates (measured using agar models) (d). Graphs show mean values ± 1 *SE*. Asterisks denote significant differences between groups

**Figure 5:**
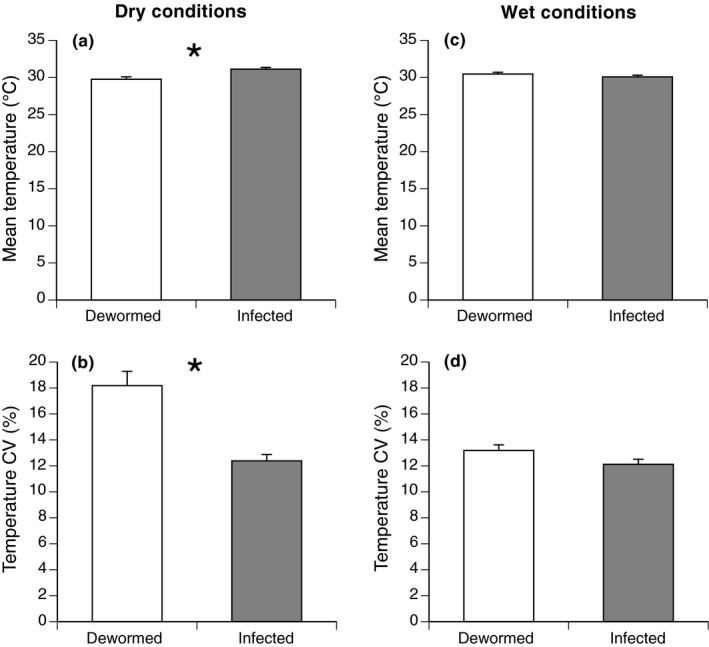
Comparison of thermal characteristics of refuge sites used by 23 radio‐tagged cane toads initially infected with *Rhabdias pseudosphaerocephala,* 12 of which had been subsequently dewormed. In dry conditions, refugia used by six infected toads were warmer (a) and had more stable temperatures (b) than those used by six dewormed toads. Under wet conditions after a period of heavy rain, five infected and six dewormed toads were found in refugia with similar thermal means (c) and variances (d). CV = coefficient of variation. Graphs show mean values ± 1 *SE*. Asterisks denote significant differences between groups

**Figure 6:**
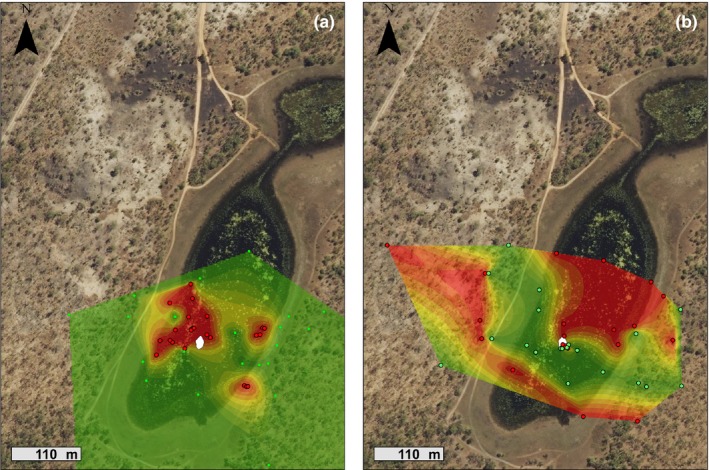
Heat map of the study site comparing diurnal refugia locations of infected and dewormed cane toads. Under dry conditions (a), infected toads (red points) used refugia closer to the remaining area of water than did dewormed toads (green points). After a period of heavy rain (b), infected toads used refugia further away from the waterbody. The small central white area shows the extent of the waterbody at the time of the study

Under dry conditions (the first 5‐day period of telemetry), the six infected toads (IC) stayed closer to water than did the six dewormed toads (Figures 4 and 6, Table 1). Agar models placed in the refugia of infected toads lost more water than those in the refugia of dewormed toads (Figure 4, Table 1). Refugia of infected toads exhibited higher mean temperatures and lower variation in temperature than did refugia of dewormed toads (Figure 5, Table 1).

Under rainy conditions (the second 5‐day period of telemetry), neither the mean distance from water nor rates of water loss by the agar models differed significantly between the refugia used by infected and dewormed toads (Figures 4 and 6, Table 1). Indeed, the heavy rain resulted in soil moisture content at refugia not differing significantly with distance from the waterbody, over a 316‐m transect (*F*_1,43_ = 2.97, *p *=* *.09), indicating that the entire local landscape had become saturated. Thermal attributes of refugia also did not differ significantly between infected and dewormed toads during this period of wet weather (Table 1).

## 4 DISCUSSION

Infection with the native‐range lungworm *Rhabdias pseudosphaerocephala* is common within the invasive range of cane toads in Australia, with these nematodes parasitizing about half the population of adult toads in our study site. Rates of lungworm infection vary seasonally and geographically in Australian toads and depend on toad body size, but high rates of infection are common (Barton, 1998; Phillips et al., 2010; Pizzatto et al., 2013). Thus, the possibility that these lungworms manipulate the behavior of their cane toad hosts suggests that any analysis of the invasion of toads through tropical Australia needs to consider the potential effects of lungworm infection not just on general toad viability (e.g., survival and growth), but also on specific behaviors (including an induced preference for wet and warm sites) that might influence the habitat selection of the invader.

The close correspondence between our field and laboratory trials and the general lack of any effect on the experimental manipulation (injection of deworming solution) in uninfected toads (procedural controls) indicate that the parasite‐related effects we have documented are real. Additionally, our data on both captive and free‐ranging adult toads verify the effectiveness of other critical assumptions of our methods (e.g., that agar models provide valid predictions of desiccation rates of adult toads and their feces). The most significant result from our study is that in virtually every trait we examined, removing lungworm infection not only was associated with a change in toad behavior, but that the direction of that change was consistent with predictions based on the hypothesis of host manipulation. That is, the ways that infected toads behaved had the effect of increasing the probable fecundity and larval survival of lungworms. However, although a link between increased fecundity and increased lifetime reproductive success seems likely, it needs to be verified to support our conclusion that manipulation is indicated (Cézilly & Perrot‐Minnot, 2010; Perrot‐Minnot et al., 2012).

Infection caused free‐ranging hosts to stay (and defecate) closer to the water, in moist habitats where the survival rates of larval parasites (and chances of encounter with another toad) are highest (Cohen & Alford, 1996; Kelehear, Webb, Hagman, & Shine, 2011; Pizzatto et al., 2013). Also, captive trials showed that infection increased rates of defecation, of moister‐than‐usual feces—again depositing larval parasites in favorable conditions for their survival. Infected and uninfected toads lost water across epidermal surfaces at a similar rate, but infected toads lost more water in their feces than did uninfected toads. This increased fecal (but not transdermal) water loss may leave infected toads with a water budget deficit, perhaps explaining why infected toads stayed closer to water.

Overall, the clear pattern from our results is that the single treatment group that maintained lungworm infections (IC; infected toads given Amphibian Ringer's solution) behaved differently than any of the other experimental groups. By seeking both warmer and wetter environments, infected cane toads increase the production of *Rhabdias* larvae and deposit them in areas that increase chances of survival and transmission to a new host. In total then, our data support the hypothesis that lungworms manipulate host behavior in ways that enhance parasite fitness. Nonetheless, although our results are supportive, future studies linking behavioral differences to parasite fitness are necessary to compellingly distinguish between manipulation and pathology.

There are two plausible alternative interpretations to the “adaptive manipulation of hosts by parasites” hypothesis:

1. These impacts are nonadaptive by‐products of illness; that is, they are part of a general “sickness behavior” syndrome (Kavaliers, Colwell, & Choleris, 2000; Klein, 2003). These lungworms do indeed induce such effects (Finnerty et al., 2017; Finnerty, P. B. 2017), but the specific changes examined in the current study seem unlikely to have arisen in this way. We would not expect general lassitude to result in a toad actively selecting warmer and wetter microhabitats, nor selectively depositing wetter‐than‐usual feces in damp sites. On the contrary, these are exactly the changes that might enhance parasite fitness.
2. These impacts reflect adaptive behavior on the part of the host, to kill the parasites within it. In keeping with that possibility, elevated temperatures of infected hosts often function in this way (“behavioral fever”: Elliot, Blanford, & Thomas, 2002). However, our laboratory trials showed that output of larvae per host was increased not reduced by the selection of higher body temperatures (at least over the short‐term). Overall, the nature and direction of these changes in behavior seem unlikely to kill or damage the pathogen or prevent its further proliferation and transmission (Kelley et al., 2012). Instead, the nature of the changes is consistent with the parasite enhancing its own fitness by subverting host behavior.

Adaptive manipulation of toad behavior may also have energetic ramifications for a host. By staying closer to a body of water and reducing movement, prey intake would be curtailed (Child, Phillips, Brown, & Shine, 2008). Combined with the costs of an upregulation of a host's immune system when parasitized (Finnerty, 2017), the adaptive manipulation of host behavior may have ramifications for a host's overall growth, performance, and viability (Finnerty et al., 2017; Finnerty, 2017).

Although our data do not allow us to unequivocally conclude that the behaviors of infected cane toads have evolved via parasite‐induced manipulation, the evidence is as strong as that often used to infer adaptation (Pérez‐Jvostov, Hendry, Fussmann, & Scott, 2015; Sternberg, Li, Wang, Gowler, & de Roode, 2013). That is, infection with *R. pseudosphaerocephala* causes behavioral shifts in the host*,* in ways that apparently enhance the survival, transmission, and reproductive output of the parasite. This is an example of the “extended phenotype” (Dawkins, 1999), whereby components of the behavior and physiology of one organism (the host) are driven by the DNA of the infective lungworm parasite. In that sense, the modification of toad behavior is part of the extended phenotype of the lungworms within it.

Fortuitously, the change in weather conditions partway through our radiotelemetry trials showed how local environmental conditions can modify the impacts of parasitism on host ecology. In a wet world, the impacts of *R. pseudosphaerocephala* were weaker. The onset of heavy rainfall resulted in all potential refugia being damp and hence eliminated the hydric difference between infected and uninfected toads. “Optimal” wetter and hotter refugium conditions were no longer scarce, and thus, infected toads could select refugia much more randomly than when conditions were drier. Given that the native‐range South and Central American home of *R. pseudosphaerocephala* (Dubey & Shine, 2008; Garreaud, Vuille, Compagnucci, & Marengo, 2009) is wet virtually year‐round (Tingley, Greenlees, & Shine, 2012; Tingley & Shine, 2011), it is possible that parasite manipulation of host hydric selection behavior might be irrelevant in such a climate. If so, this aspect of the parasite–host manipulation at Leaning Tree Lagoon might have arisen during the course of the cane toad's invasion of Australia. A majority of the invaded range of cane toads in Australia experiences a wet–dry tropical climate, with monsoonal rainfall only between November and April (Shine & Brown, 2008). Given the aridity of the remaining 6 months of the year, manipulating a host to remain close to (and defecate on) damp soil would markedly increase survival of infective larvae and hence transmission to another host. As a result, this proposed parasite–host manipulation may be seasonal in its impact (as suggested by the breakdown of the effect after heavy rainfall) within most of the toad's Australian range.

It would be of great interest to establish whether lungworms have similar effects on cane toad behavior in other parts of the toad's extensive range (Thomas et al., 2010). As noted above, long dry periods are rare in the native ranges of the host (Dubey & Shine, 2008; Garreaud et al., 2009), suggesting that behavioral manipulation of the host may not be as important in providing infective stage larvae with optimal wetter and hotter conditions for survival and transmission. The same might be true in parts of the wet tropics of eastern Australia, where rainfall occurs year‐round (Pepler, Coutts‐Smith, & Timbal, 2014). Thermal manipulation of the host is unlikely to be significant across warmer parts of the toad's range (in both Australia and elsewhere) but may become important at the southern front of the toad invasion in eastern Australia (McCann, Greenlees, Newell, & Shine, 2014; Seabrook, 1991). Likewise, severely arid conditions in the Western Australian invasion range of the cane toad may impose stronger benefits to parasites that induce toads to select moist sites and produce feces with higher water content.

Given that lungworms appear to manipulate several aspects of cane toad behavior and physiology, by what mechanisms are such effects achieved? How can a parasite without direct contact to a host's brain or neural tissue (i.e., in the lungs) alter host behavior (Biron et al., 2006)? There are various possibilities. Pathological effects on the host's lungs and gut are minor (Finnerty, 2017), suggesting that behavioral changes may be mediated instead via the neuronal system of the host (i.e., through neurotransmitters, neuromodulators, and hormones; Schmid‐Hempel, 2011). Macroparasites (like *R. pseudosphaerocephala*) may be able to produce enough neuromodulators to directly affect host behavior (Adamo, 2003; Lafferty & Shaw, 2013; Schmid‐Hempel, 2011). For example, trematodes (*Euhaplorchis californiensis*) infecting killifish (*Fundulus parvipinnis*) secrete endorphins, peptides, and fibroblast growth factors associated with neuropathology that result in increased swimming near the water surface, promoting the transmission of the parasite to the final (definitive) avian host (Lafferty & Morris, 1996). Investigation into the biochemical pathways that induce changes in a host's behavior would be a useful avenue for future research.

Our results suggest that the ways in which parasites can manipulate host behavior can be subtle and independent of obviously pathological consequences of infection (e.g., terminal investment in reproduction and sickness behavior induced by inflammation). The lifecycles of parasites are often intimately matched to existing host behaviors (e.g., gravid female *Dracunculus* migrate to the limbs of the raccoon [*Procyon lotor]* host and shed eggs as the host dabbles in water). However, our study shows that it is also possible for parasites to manipulate minor aspects of host behavior (e.g., selection of sites used for shelter or defecation), in ways that may significantly enhance parasite fitness. Experimental deworming may be a useful methodology to determine the generality of such subtle modifications in host–parasite systems. It would also be of great interest to determine what costs such manipulations impose on the host and the mechanistic pathways that underlie these behavioral shifts.

Although our correlative results support the hypothesis that lungworms manipulate toad behavior, more study is required to test that conclusion. Studies on host manipulation by parasites are in their infancy, and besides a general lack of understanding of mechanisms, it is often difficult to distinguish between adaptive manipulations and pathological side effects (Herbison, 2017). For example, behavioral fever is a common response to pathogen infection in ectotherms (Rakus et al., 2017). We observed that infected toads selected higher body temperatures that resulted in increased rates of egg production by their lungworms. Ideally, we need to ascertain that this increased egg production actually increased fitness of the parasite. The same holds true for other behavioral differences we observed between infected and uninfected hosts; the impact of behaviors on lifetime fitness of the parasite (and host) must be established to provide compelling evidence that manipulation is occurring (Cézilly & Perrot‐Minnot, 2010; Perrot‐Minnot et al., 2012). Identifying the mechanisms by which parasites manipulate their hosts remains a formidable challenge, but advances in gene expression techniques hold promise in this respect (Feldmeyer et al., 2016; Herbison, 2017).

## CONFLICT OF INTEREST

None declared.

## AUTHOR CONTRIBUTIONS

PF, GB, and RS conceived the ideas and designed methodology; PF and GB collected and analyzed the data. All authors contributed to writing the manuscript and gave the final approval for publication.

## DATA ACCESSIBILITY

Data are deposited in the Dryad repository under Provisional DOI: https://doi.org/10.5061/dryad.3p5r6.
