# Supplementary material for: Survival of the feces: Does a nematode lungworm adaptively manipulate the behavior of its cane toad host?
Source: Ecol Evol. 2018 Apr 15;8(9):4606–18. doi: 10.1002/ece3.3870 (PMC5938457; doi:10.1002/ece3.3870)
Supplement: Supplementary file 1 [file ECE3-8-4606-s001.docx]

**Supplementary Information**

**Survival of the faeces: does a nematode lungworm adaptively manipulate the behaviour of its cane toad host?**

**Patrick B. Finnerty*, Richard Shine and Gregory P. Brown**

**Calibration of agar models of toads and their faeces**

In the same way that agar models have been used to estimate desiccation rates of adult and metamorph toads (Schwarzkopf and Alford 1996, Navas and Araujo 2000, Child *et al.* 2008), we used agar models to estimate desiccation rates of toad faeces and adult cane toads in the wild. Agar (Sigma A7002) solution was boiled (2% agar to 98% water) then poured into a baking tray and allowed to set before cutting out individual models. For the comparison with toad faeces, we used 30 × 10 × 10 mm blocks of agar. For the comparison with adult toads, we used larger 30 × 30 × 10 mm blocks with a thermal data-logger embedded in the lower surface of each larger model.

We calibrated desiccation rates of the small agar models against samples of faeces supplied by captive toads of all four treatment groups (IC, ID, NC, ND). Fresh faecal samples (*n* = 5) were split in half, weighed and placed on top of 60 g of heat-sterilized sand in open 25ml plastic vials. The same was done with 5 agar models of faeces. From each pair of vials, the sand in one was kept damp (at constant weight) whereas the other remained dry. Vials were kept at 22.9 ± 1.8°C (average humidity 52%) for three days, then re-weighed to compare desiccation rates of faeces versus agar models.

To estimate hydric conditions within refugia used by radio-tagged toads in the field, we constructed agar models designed to desiccate at a similar rate as a toad (Schwarzkopf and Alford 1996, Navas and Araujo 2000, Child *et al.* 2008). To calibrate desiccation rates of these agar models against adult toads, we weighed 10 captive toads (of random treatment groups) and 10 large agar models (to the nearest 0.001 g), and placed them individually in 5 L plastic containers. Half were placed in a 'wet treatment' (container lined with damp paper towel) and half in a 'dry treatment' (container without damp paper towel). Each container had a mesh lid and two large mesh apertures on the sides to allow airflow. After 24 hours at 32.5 ± 0.03°C, we re-weighed all toads and agar models, and calculated % water loss.

**Agar model calibration results**

Desiccation rates were higher in dry conditions for agar models, toads, and toad faeces (Table SI(i), Fig. SI(i)), with desiccation rates of faecal samples and toads similar to those of the equivalent-sized agar models in both dry and wet conditions (Table SI(i)). Thus our agar models produced realistic representations of desiccation rates of both toads and their faeces.

**Table SI(i).** Effects of treatment (dry vs wet), object (adult toad / toad faeces vs agar representations), and their interaction on desiccation rates of agar models, cane toads and toad faeces over a 72-hour period. Significant values (*P* < 0.05) are shown in boldface font. Both types of agar model lose water at the same rate as the object they were designed to represent.

| Test | Dependent variable | Independent variable | *DF* | *F* | *P* |
| --- | --- | --- | --- | --- | --- |
| *Faecal vs agar model* | |  |  |  |  |
|  | Desiccation rate | Faeces or agar | 1,14 | 0.07 | 0.88 |
|  | Desiccation rate | Dry or wet | 1,14 | 318.41 | **<0.01** |
|  | Desiccation rate | Faeces or agar*Dry or wet | 1,14 | 0.49 | 0.5 |
| *Toad vs agar model* | |  |  |  |  |
|  | Desiccation rate | Toad or agar | 1,16 | 0.08 | 0.78 |
|  | Desiccation rate | Dry or wet | 1,16 | 351.5 | **<0.01** |
|  | Desiccation rate | Toad or agar*Dry or wet | 1,16 | 0.95 | 0.34 |

**Figure SI(i)** Comparison of desiccation rates (% water loss after 72 hrs) of (a) faecal samples vs and agar models of faeces, and (b) adult toads vs agar models of toad models under dry and wet conditions. Blank bars represent dry conditions, grey bars represent wet conditions. Graphs show mean values ± 1 SE, Bars with the same alphabetical superscript are not significantly different from one another (*P* > 0.05).**Methods to evaluate potential bias in visibility of different UV powder colours**

To check that all UV fluorescent colours were equally visible (an assumption inherent in our method of using coloured powder to locate faecal samples in the field), we mixed 0.10 mL dose of UV fluorescent powder solution with 1.5 g of moist, dark mud (gathered from Leaning Tree Lagoon) to mimic toad faeces. The resulting mixture was placed in a small closed 1.5 mL vial. A row of four differently coloured vials (green, orange, pink, and yellow) were placed in a random assortment in short grass (10–30 mm) at 2-m intervals up to 16 m from the point where the observer was standing with the UV torch. We stood 2 m from the first set of vials and shone the UV light across each row of vials, recording if they were visible or not. We repeated this test for each row until the vials could no longer be seen. All colours were equally visible over 14 m (but no further; this was at the end of the range of the UV torch).

To determine if each colour was equally detectable in different habitat types (flat, dry soil, short grass, long grass, and mixed habitat containing short and long grass, leaf litter and small branches) we threw seven vials of each colour over a shoulder all at the same time (approximately 8.5 m), turned back around, and used the UV light to try and locate each vial. All four fluorescent pigments used were equally retrievable from the four habitat types tested over four retrieval trials on different nights (Table SI(ii), Fig. SI(ii)). Analysis revealed that vials were more easily seen in some habitats than others (Table SI(ii)); retrieval rates were lower in long grass and mixed habitats.

**Table SI(ii).** Results of statistical analyses of data on retrieval rates of four different fluorescent colour pigments mixed with 1.5 g of mud over four habitat types. The table provides results from a nominal logistic fit model. Significant values (*P* < 0.05) are shown in boldface font.

| Variable | *df* | χ^2^ | *P* |
| --- | --- | --- | --- |
| Colour | 3 | <0.01 | 1 |
| Habitat | 3 | 15.28 | **<0.01** |
| Approximate distance thrown (m) | 1 | 0.01 | 0.90 |
| Colour*Habitat | 9 | 1.27 | 0.99 |

**Figure SI(ii).** All fluorescent pigments used were equally retrievable from all four habitat types over four retrieval trials. A lower retrieval rate occurred in long grass and mixed habitats. Different bar patterning denotes different habitats.
